# Supplementary material for: Determinants of glycemic control among persons living with type 2 diabetes mellitus attending a district hospital in Ghana
Source: PLoS One. 2024 Nov 25;19(11):e0308046. doi: 10.1371/journal.pone.0308046 (PMC11588282; doi:10.1371/journal.pone.0308046)
Supplement: Attachment — Submitted filename: Response to Reviewers.pdf [file pone.0308046.s002.pdf]

Shai-Osudoku District Hospital
P.O Box DD1
Dodowa - Greater Accra Region
Ghana
15th October 2024

The Editor,
PLOS ONE

Dear Sir,

<u>Response to reviewer's comments Manuscript PONE-D-24-28656</u>

We write to officially respond to the reviewer's comments on our manuscript entitled: "**Determinants of Glycemic Control among persons living with Type 2 diabetes mellitus attending a District Hospital in Ghana"** to be considered for publication in PLOS ONE Journal as an original article.

Attached to this cover letter is a point-by-point response to all the comments and corrections made by the editor with added information, clarifications and corrections as suggested.

Thank you for receiving our manuscript and considering it for review. We appreciate your time and look forward to hearing favourably from you.

Sincerely,

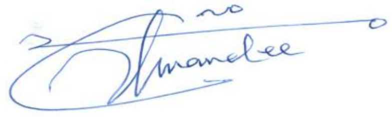

Corresponding author
Alexander Adjei
Email: caesar306@yahoo.com
Tel: +233 24 472 7070

Response to Reviewer #2 comments

General Comments

1. Title and Abstract: The title accurately reflects the content of the study. The abstract is well-written, providing a concise summary of the study's objectives, methods, results, and conclusion. However, consider adding more details about the statistical methods in the abstract for clarity.

   Response: Thanks for this comment. We believe that statistical methods in the abstract are appropriate. A detailed description has been provided in the data analysis part of the methods section of the paper.

2. Introduction: The introduction provides a good background on the global and local burden of diabetes mellitus. The rationale for the study is clearly articulated, and the objectives are well-defined. However, the introduction could benefit from more recent references to strengthen the context of the study.

   Response: Five more current papers have been added to this section and these are reference numbers 10, 13, 29, 33 and 34 on pages 4, 5 and 16.

3. Methods: The methods section is detailed and appropriate for the research question. The study design, sampling method, and data collection procedures are well-explained. The statistical analysis methods are appropriate, but the handling of potential confounders should be described in more detail.

   Response: Multivariate logistic regression accounts for potential confounders, which the study design does not address. This has been acknowledged under the limitation section of the paper on page 18.

4. Results: The results are clearly presented with appropriate use of tables and figures. However, some results are repeated in the text that are already shown in tables, which may be unnecessary. Consider summarizing key points in the text and referring to the tables for detailed results.

   Response: The content on Table 1 provides a summary of the pertinent variables regarding the background of this study. We believe this to be appropriate.

   The wording on Table 2 summarizes only three out of ten variables, while that on Table 3 outlines the significant factors derived from the multivariate logistic regression.

5. Discussion: The discussion appropriately interprets the results in the context of existing literature. The implications of the findings are well-discussed, but the discussion of the study's limitations could be expanded, especially regarding the potential biases in self-reported data and the cross-sectional nature of the study.

   Response: The potential biases and limitations of the study design have been emphasized in the limitation section on page 18.

6. Conclusion: The conclusion is concise and highlights the key findings of the study. It appropriately emphasizes the need for improved dietary management among T2DM patients.

   Response: Thanks for the comment.

7. References: The references are generally well-chosen and relevant. However, some references are relatively old, and the inclusion of more recent studies could enhance the discussion.

   Response: Five new references have been added as stated above.

<u>Specific Comments</u>

1. Clarity and Readability: Some sentences are complex and could be simplified for better readability. For example, the sentence "The cornerstone of diabetes management is glycemic control, a complex process with different contributing factors" could be simplified to "Glycemic control is central to diabetes management, but it is influenced by various factors."

   Response: Thanks for the comment and the statement has been revised as suggested under the background section of the abstract on page 2.

2. Consistency in Terminology: Ensure that terms such as "glycemic control" and "T2DM" are used consistently throughout the manuscript. For instance, "glycemic control" is sometimes referred to as "blood glucose control" or "HbA1c levels," which could confuse readers.

   Response: Thanks for your comment. We carefully considered the terminologies used to ensure they accurately convey the intended message.

3. Statistical Analysis: The choice of statistical tests is appropriate, but the manuscript could benefit from a more detailed explanation of the multivariate logistic regression model, particularly how variables were selected for inclusion in the model.

   Response: This has been done by including the statement "A p-value of 0.05 from the Chi-square test was used for the multivariate logistic regression" under data management and analysis on page 10.

4. Figures and Tables: Ensure that all figures and tables are clearly labeled and referenced in the text. Some tables may benefit from additional footnotes explaining abbreviations or providing context.

   Response: Footnotes have been added to tables 1 and 2 on BMI and MGL as follows; "BMI - Body mass index, MGL - Morisky Green Levine medication adherence scale" on pages 12 and 14.

5. Limitations: The limitations section is brief and could be expanded to discuss the potential impact of selection bias, the accuracy of self-reported data, and other factors that may have influenced the study's findings.

   Response: The limitation section has been emphasised to reflect these raised comments on recall bias as follows; "The findings of this study need to be considered in the context of this potential bias due to self-reported data".

   On possible confounders, as follows; "Confounding factors like anemia and hemoglobin disorders may not be evenly distributed, leading to bias and misinterpretation. Multivariate logistic regression analysis helps to control for these confounding variables" on page 18.

Thanks for the recommendations.
